# Mössbauer Spectroscopy of Iron Carbides: From Prediction to Experimental Confirmation

**DOI:** 10.1038/srep26184

**Published:** 2016-05-18

**Authors:** Xing-Wu Liu, Shu Zhao, Yu Meng, Qing Peng, Albert K. Dearden, Chun-Fang Huo, Yong Yang, Yong-Wang Li, Xiao-Dong Wen

**Affiliations:** 1 State Key Laboratory of Coal Conversion, Institute of Coal Chemistry, Chinese Academy of Sciences, Taiyuan, 030001, P.R. China; 2 National Energy Center for Coal to Clean Fuels, Synfuels China Co., Ltd, Huairou District, Beijing, 101400, P.R. China; 3 University of Chinese Academy of Sciences, No. 19A Yuquan Road, Beijing, 100049, P.R. China; 4 Department of Mechanical, Aerospace and Nuclear Engineering, Rensselaer Polytechnic Institute, Troy, NY 12180, USA; 5 Department of Physics, Berea College, Berea, KY 40403, USA

## Abstract

The Mössbauer spectroscopy of iron carbides (α-Fe, γ'-FeC, η-Fe_2_C, ζ-Fe_2_C, χ-Fe_5_C_2_, h-Fe_7_C_3_, θ-Fe_3_C, o-Fe_7_C_3_, γ'-Fe_4_C, γ''-Fe_4_C, and α'-Fe_16_C_2_) is predicted utilizing the all electron full-potential linearized augmented plane wave (FLAPW) approach across various functionals from LDA to GGA (PBE, PBEsol, and GGA + U) to meta-GGA to hybrid functionals. To validate the predicted MES from different functionals, the single-phase χ-Fe_5_C_2_ and θ-Fe_3_C are synthesized in experiment and their experimental MES under different temperature (from 13 K to 298 K) are determined. The result indicates that the GGA functional (especially, the PBEsol) shows remarkable success on the prediction of Mössbauer spectroscopy of α-Fe, χ-Fe_5_C_2_ and θ-Fe_3_C with delocalized d electrons. From the reliable simulations, we propose a linear relationship between B_hf_ and μ_B_ with a slope of 12.81 T/μ_B_ for iron carbide systems and that the proportionality constant may vary from structure to structure.

Numerous iron carbides have been reported in chemical and metallurgical literatures ranging in composition from FeC to Fe_4_C^1^,^2^. Owing to their outstanding mechanical, catalytic, and magnetic properties with the presence of carbon atoms, iron carbides have been widely used in both practical industries^3^,^4^ (for instance, Fischer-Tropsch synthesis (FTS) for liquid fuels, carbon steel materials and carbon nanotubes synthesis, etc.), and fundamental materials science^5^.

Cementite (θ-Fe_3_C) is a well-known carbide that is a component of pearlite in carbon steel and acts as a catalytically active phase of carbon nanotube synthesis. In the Fe-based FTS, ε-Fe_2_C, ε’-Fe_2.2_C, Fe_7_C_3_, χ-Fe_5_C_2_, and θ-Fe_3_C have been observed^12^, and there are complicated phase transitions under reaction conditions. The high pressure phase of Fe_7_C_3_ was proposed to be formed in the Earth’s inner core^13^. γ'-FeC was studied theoretically by Lee^14^ and observed experimentally by Cusenza *et al.*^15^ using pulsed laser deposition, which has a cubic NaCl-type structure with 4 formula units per unit cell.

To identify different iron carbides is a long-standing challenge due to the metastable phases with flat potential energy surfaces and light C elements. Characterization techniques such as thermomagnetic analysis (TMA)^16^,^17^,^18^, X-ray diffraction (XRD)^19^,^20^, and Mössbauer spectroscopy (MES)^21^ have been widely used to characterize iron carbides. TMA is based on the observation that a ferromagnetic phase loses its ferromagnetism at the Curie temperature^16^,^17^, which only provides the Curie point without any other structural information^18^. XRD is a common technique to identify phases in materials science. Most of the iron carbides have distinct XRD except for octahedral carbides (ε-Fe_2_C, ε’-Fe_2.2_C, and ε-Fe_3_C) which have similar crystallographic structures^20^. However, one should note that XRD patterns are only representative of bulk compositions. The formed carbides are usually in small particles (nano-scale) with metastable and distorted crystal structures^5^, which results in peak broadening and intensity lowering. Moreover, amorphous and surface layers are invisible to XRD.

MES is an excellent characterization technique for iron-based materials, especially for the identification of phases, such as nanoparticles that contain poorly crystallized, microcrystalline, or amorphous phases^22^,^23^. In general, the applications of MES on iron containing materials fall in one of the five categories, such as phase identification (iron oxides or iron carbides), determination of oxidation states, structure information, particle size determination, and kinetics of bulk transformations^24^. The hyperfine parameters extracted from spectral analysis can yield detailed local electronic, magnetic, structural, and chemical information on Fe atoms in samples^25^,^26^. For example, the Mössbauer spectrum of χ-Fe_5_C_2_ is usually fitted with three sextets that correspond to the three crystallographic sites of the structure that may be occupied by the iron atoms, thus leading to a different atomic environment for each of them^27^. Moreover, from the relative intensities of the three sextets used for the fit and considering equal recoil-free factors for the corresponding species, it is possible to determine the respective occupation of these sites by iron. Similarly, θ-Fe_3_C has two chemically different iron positions, each characterized by a sextet in the spectra.

Up to now, although MES is widely used to identify iron carbides, the Mössbauer parameters including quadrupole splitting (QS), isomer shift (IS), and magnetic hyperfine field (B_hf_) of θ-Fe_3_C and χ-Fe_5_C_2_ are hitherto unambiguous. The Mössbauer spectrum of NaCl-type γ'-FeC, η-Fe_2_C, ζ-Fe_2_C, h-Fe_7_C_3_, o-Fe_7_C_3_, γ'-Fe_4_C, γ''-Fe_4_C, and α'-Fe_16_C_2_ are not reported in both experiment and theory owing to the metastable phases or no pure single phases in experiment, as well as lacking a systematic benchmark on theoretical methods.

The Mössbauer parameters are linked to the electron/spin density and spin orbital coupling, which can be obtained from Density Functional Theory (DFT)^26^,^28^. DFT is, in principle, an exact theory. However, the major problem with DFT is that the exact functional for exchange and correlation is not known leading to a hierarchy of approximations; the local-density approximation (LDA), generalized gradient approximations (GGA), meta-GGA, hybrid functionals, etc. The LDA and GGA usually slightly underestimate and overestimate equilibrium lattice constants by about 1%, respectively. Other equilibrium properties, such as phonon frequencies, magnetism, bulk moduli, etc. are sensitive to the lattice constant. The meta-GGA with the orbital kinetic-energy densities, provides better accuracy on properties over a range of systems, and does not dramatically improve the lattice constants. Hybrid DFT significantly improves the descriptions of d- or f-electron systems, generally predicting the correct insulating and magnetic behavior in Mott insulating cases.

Currently, a comprehensive theoretical simulation for MES parameters of iron carbides via DFT is not readily available. Furthermore, a fundamental understanding of the relationship between MES and the electronic and geometric structures is highly desirable. Most importantly, it is necessary to know whether the functionals in DFT are suitable to calculate Mössbauer parameters, which requires an accurate description of the wavefunctions also in the core region of a Mössbauer atom.

In this work, the Mössbauer parameters of α-Fe, θ-Fe_3_C, and χ-Fe_5_C_2_ are initially calculated with different approaches and compared with current experimental data to confirm/benchmark a reliable approach. In addition, the Mössbauer parameters of γ'-FeC, η-Fe_2_C, ζ-Fe_2_C, h-Fe_7_C_3_, o-Fe_7_C_3_, γ'-Fe_4_C, γ''-Fe_4_C, and α'-Fe_16_C_2_ are theoretically predicted. The nearly single-phase χ-Fe_5_C_2_ and θ-Fe_3_C are prepared in experiment. Based on the data obtained, the relationship of hyperfine parameters with magnetic moment and the effect of C atoms on hyperfine fields of Fe are investigated and discussed in order to make a calibration between magnetic properties and structures on iron carbides.

## Results and Discussion

### Structures and Their Stability

**Figure 1:**
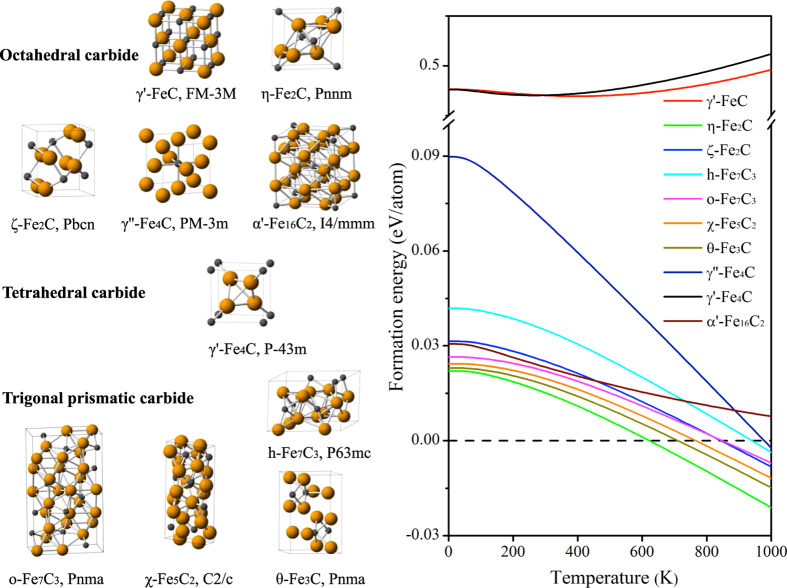
The crystal structure of iron carbides classified by octahedral, tetrahedral, and trigonal prismatic carbide, respectively, as well as the formation energy as a function of temperature (reference to α-Fe and graphite).

These carbides can be classified as octahedral carbides, tetrahedral carbides, and trigonal prismatic carbides according to the sites occupied by the carbon atoms^29^,^30^. As shown in Fig. 1, the octahedral carbides include γ'-FeC, α'-Fe_16_C_2_, η-Fe_2_C, ζ-Fe_2_C, and γ''-Fe_4_C. In addition, γ'-FeC and γ''-Fe_4_C consist of fcc-Fe sublattices with octahedral C interstitial atoms. η-Fe_2_C and ζ-Fe_2_C have hexagonal close packing Fe sublattices. α'-Fe_16_C_2_ is the isostructural with α'-Fe_16_N_2_ which consists of bcc-Fe sublattices with N interstitial atoms occupying the octahedral sites. The second type is trigonal prismatic carbide with members h-Fe_7_C_3_, o-Fe_7_C_3_, χ-Fe_5_C_2_ and θ-Fe_3_C, of which the C atoms are located in the trigonal prismatic sites of distorted hexagonally closed packed structure of Fe atoms. The last type is tetrahedral carbide, γ'-Fe_4_C. The detailed crystal structural information is listed in Supporting Information (Table S1). The average Fe-C distance is around 1.90 Å in these carbides, which falls within the expected range of Fe-C distances for many structures (see Figure S2 and S3) in the Inorganic Crystal Structure Database (ICSD). The nearest C-C distance is varying from 2.60 Å to 3.50 Å, indicating that there are no C-C dimers in iron carbides.

To investigate the thermodynamic properties of these iron carbides, we calculate the formation energy with zero point energy corrections of iron carbides (reference to α-Fe plus graphite) at various temperature (from 0 to 1000 K), as plotted in Fig. 1. One can see that the reactions forming all iron carbides from α-Fe and graphite are endothermic at low temperature zone (<600 K), which means the carbides could decompose into α-Fe and graphite during tempering reactions. The computed phonons without imaginary modes indicate all of these carbides are dynamically stable, indicating that the carbides given are located in minima of the “flat” potential surface. In fact, the synthesis approach is the kinetic effect on forming carbides, such as extreme condition synthesis (high pressure) for Fe_7_C_3_ and FeC, and gas carburization for Fe_2_C, Fe_5_C_2_, and Fe_3_C, etc. As computed from the formation energy from the reaction, (x/y)Fe + 2CO = (1/y)Fe_x_C_y_ + CO_2_, one can see that for most of the iron carbides considered, the process is theoretically favorable (see Figure S1). The metastable property causes the challenge of Mössbauer characterization for iron carbides in experiments.

### Experimental Mössbauer Parameters of χ-Fe_5_C_2_ and θ-Fe_3_C

**Figure 2:**
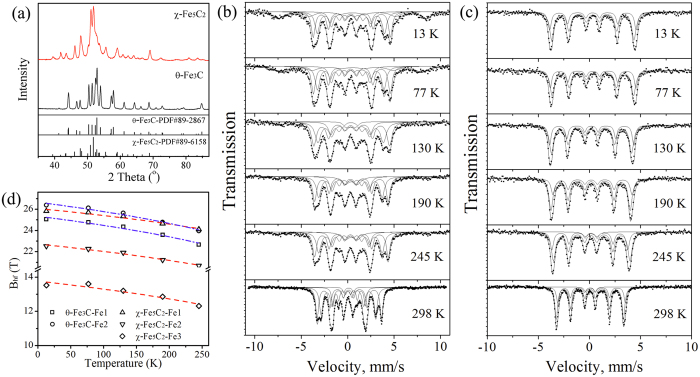
(**a**) XRD patterns of χ-Fe_5_C_2_ and θ-Fe_3_C synthesized; Mössbauer spectrum of χ-Fe_5_C_2_ (**b**) θ-Fe_3_C (**c**) obtained at 13, 77, 130, 190, 245 and 298 K, respectively. The solid lines are least-squares fits to the Mössbauer spectra; (**d**) Temperature dependence of the Hyperfine field of the Fe atoms for χ-Fe_5_C_2_ and θ-Fe_3_C. The dash dot lines are fitted using a power law function.

In order to obtain reliable and reasonable experimental Mössbauer parameters of χ-Fe_5_C_2_ and θ-Fe_3_C in the study, the nearly single-phase θ-Fe_3_C and χ-Fe_5_C_2_ are prepared by gas carburization. Fig. 2(a) presents the XRD patterns of χ-Fe_5_C_2_ and θ-Fe_3_C. All peaks are in good agreement with the standard spectrum (JCPDS no.: 89-6158 and 89-2867), which indicates that the contents of χ-Fe_5_C_2_ and θ-Fe_3_C are high enough in these samples.

The experimental spectra of χ-Fe_5_C_2_ and θ-Fe_3_C together with their best fits are shown in Fig. 2(b,c), respectively. One doublet and three sextets are used to fit the room temperature Mössbauer spectra of χ-Fe_5_C_2_. The parameters of three sextets are in agreement with reported literature^18^,^27^,^31^,^32^, which corresponds to the three types of Fe (Fe1, Fe2 and Fe3) in the unit cell of χ-Fe_5_C_2_, respectively. As reported in previous studies, two sextets are needed to fit the Mossbauer spectra of θ-Fe_3_C. The parameters of the room temperature spectrum of θ-Fe_3_C consist of two sextets and one doublet. The B_hf_ of Fe1 in θ-Fe_3_C is 20.9 T which is consistent with 20.8 T reported by Le Caer^32^, but the B_hf_ of Fe2 is 19.4 T, a little lower than 20.6 T. In addition, it is noted that there are superparamagnetic (SPM) species in the sample θ-Fe_3_C due to the doublet. Fig. 2(d) shows the B_hf_ of χ-Fe_5_C_2_ and θ-Fe_3_C versus temperature. One can see that the hyperfine fields of iron carbides decrease from 13 K to 298 K. The temperature dependence of hyperfine field can be described by the power law function, B_hf_(T) = B_hf_(0 K)(1 − T/T_c_)^β^, where T_c_ is the curie temperature (for χ-Fe_5_C_2 _T_c_ = 538 K and for θ-Fe_3_C T_c_ = 483 K^33^) and β is called a critical exponent (for mean field theory β = 0.5). The hyperfine fields of χ-Fe_5_C_2_ and θ-Fe_3_C at 0 K can be extrapolated from the measurements, which is used to compare with the current theoretical values since the Mössbauer parameter simulation is implemented at 0 K and 0 atm without thermal effects on the MES. Next, we move on to the prediction of the Mössbauer parameters.

### Simulated and Predicated Mössbauer Spectrocopy

Mössbauer parameters of α-Fe, χ-Fe_5_C_2_ and θ-Fe_3_C are calculated using the procedure described below. Firstly, it is of significance to know which functional is suitable to calculate Mössbauer parameters of iron carbides. Therefore, a systematic comparison of the computational methods is performed here. The performance of the methods is justified by comparing the Mössbauer parameters obtained from the current experimental data.

The calculated and experimental hyperfine parameters (0 K) of α-Fe, χ-Fe_5_C_2_, and θ-Fe_3_C with the different approaches are shown in Table 1. Fe and iron carbides fall into the regime of correlated metals. Interestingly, from our calculations, one can see that the prediction from the standard functional agrees well with the experimental Mössbauer parameters. However, GGA + U gives bad predictions on the hyperfine fields when U is set to 2.5, 3.5, and 4.5 eV. Note that the empirical parameter U is material-dependent and must be carefully chosen. Hybrid functionals with α = 0.25 (25% HF component) and 0.1 (10% HF component) seriously overestimate all of the Mössbauer parameters. As expected, when α is set to 0.01 (1% HF, close to GGA functional), the value of hyperfine fields are in good agreement with the experimental value. Therefore, only HF with α = 0.01 as well as LDA, GGA, and meta-GGA are discussed next.

In order to evaluate these functionals, we calculated the relative errors of IS (mm/s) and B_hf_ (T) of the three substances. A negative value for the relative error implies the underestimation of the experimental value. From Table 2, GGA-PBE, GGA-PBEsol, and HF (α = 0.01) give very good predictions on the B_hf_ of α-Fe, χ-Fe_5_C_2_, and θ-Fe_3_C with the relative error less than 10.1 %. For instance, the B_hf_ of α-Fe calculated by GGA-PBE, GGA-PBEsol, and HF (α = 0.01) are 32.7, 31.8, and 33.5 T, respectively, which are very close to the experimental value of 33 T. However, the hyperfine fields are underestimated by −5.5% and −39.4% in LDA and meta-GGA, respectively. In addition, GGA-PBEsol gives the best estimation of the Isomer shift for θ-Fe_3_C and χ-Fe_5_C_2_. The calculated ISs for Fe1 of θ-Fe_3_C and χ-Fe_5_C_2_ are 0.28 and 0.36 mm/s, which are lower than the experimental value 0.33 and 0.39 mm/s by about −15.2% and −7.7%, respectively. However, the remaining methods give lower IS values for θ-Fe_3_C (0.18 ~ 0.25 vs. 0.33 mm/s for Fe1) and χ-Fe_5_C_2_ (0.16 ~ 0.23 vs. 0.35 mm/s for Fe2). In conclusion, the comparison between experiment and theory immediately reveals that the Mössbauer parameters calculated by GGA-PBEsol are closer to the experimental values, which can give good descriptions on both the electronic and magnetic properties of α-Fe, χ-Fe_5_C_2_, and θ-Fe_3_C.

As well known^34^, the first (LDA), second (GGA), and third generation functional (meta-GGA) fail to predict the so-called correlated electrons of 3d transition metal oxides (strongly correlated insulators). One has to go beyond LDA/GGA and use methods like hybrid functionals and DFT + U to describe the correlated insulators. The origin of the issues with the hybrid functional and the DFT + U approximations is associated with overestimating localized electrons in the correlated metallic system compared with a correlated insulator. There is presently no means to ‘dynamically’ screen either the effective Hubbard U in DFT + U or the HF component in hybrid functionals “on the fly”. We propose that the two approximations are missing key physics in the correlated metal regime, as we have pointed out in our previous work on actinide materials and f electron systems^35^,^36^.

After a systematic comparison of the computational methods, it is found that the PBEsol method gives the best agreement with experimental MES of α-Fe, χ-Fe_5_C_2_, and θ-Fe_3_C. We recommend PBEsol for both quantitative prediction and qualitative analysis of hyperfine parameters of iron carbides. In principle, PBEsol is a revised PBE that improves equilibrium properties of densely-packed solids and their surfaces. Here, it also improves the magnetic properties.

**Figure 3:**
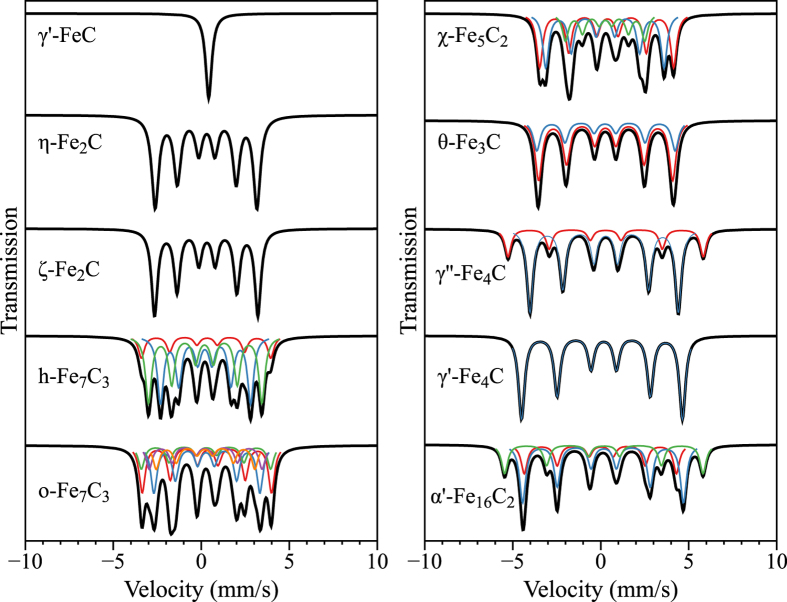
The predicted Mössbauer spectroscopy of γ′-FeC, η-Fe_2_C, ζ-Fe_2_C, h-Fe_7_C_3_, o-Fe_7_C_3_, χ-Fe_5_C_2_, θ-Fe_3_C, γ′-Fe_4_C, γ″-Fe_4_C, and α′-Fe_16_C_2_.

Using PBEsol, the Mössbauer parameters and spectrums of γ'-FeC, η-Fe_2_C, ζ-Fe_2_C, h-Fe_7_C_3_, o-Fe_7_C_3_, γ'-Fe_4_C, γ''-Fe_4_C, and α'-Fe_16_C_2_ are predicted and shown in Table 3 and Fig. 3. These theoretical values for the MES of iron carbides are important and very useful to identify and detect iron carbides experimentally in the future. In Table 3, we can see that γ'-FeC has only one set of hyperfine parameters, and QS and B_hf_ are 0 mm/s and 0 T, respectively. Which means that γ'-FeC is paramagnetic and has one singlet in Mössbauer spectra. It is interesting that two structures of Fe_2_C have almost the same hyperfine parameters due to the similar geometric structures with space group *Pnnm* (58) and *Pbcn* (60) respectively. It is impossible to distinguish them by using MES. For h-Fe_7_C_3_ and o-Fe_7_C_3_, as well as γ'-Fe_4_C and γ''-Fe_4_C, it is easy to distinguish among them by using MES, although they have the same formula. The computed Mössbauer parameters for these iron carbides can be treated as references to identify phases in experiment in the future and provide the fitting database.

### Relationship of B_hf_ with Fe/C ratio

**Figure 4:**
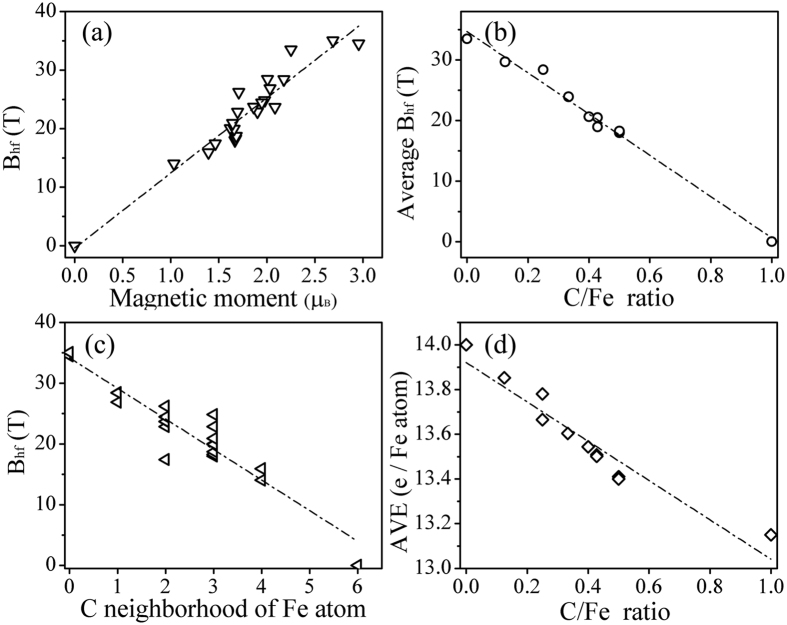
(**a**) Hyperfine fields in iron carbides as a function of magnetic moment of Fe atoms. (**b**) Average hyperfine fields in iron carbides as a function of C/Fe ratio, (**c**) The hyperfine field as a function of C atoms in the neighborhood of a single Fe atom, (**d**) Average valence electrons (AVE) of iron carbides as a function of C/Fe ratio.

The linear relationship between the magnetic hyperfine field (B_hf_) and the magnetic moment (μ_B_) in bulk has been found for many years^37^. The proportionality constant of this relationship of some iron compounds (12.9 T/μ_B_ for Fe_75_Si_15_B_10_^38^, 189.3 T/μ_B_ for RuFeSi_0.5_ 39, 9.5 T/μ_B_ for Fe_3_Sn_2_ 40) has been reported. However, as Dubiel mentioned, there is not one definite and universal constant to rescale B_hf_ into μ_B_, which depends on the compound given or even on the composition^41^. It is also interesting to extract certain relationships from the computed data for iron carbides, which is of significance to design Fe-based catalysts and materials. From Fig. 4(a), one can see that the hyperfine field and the magnetic moment of iron atoms in different iron carbides have a good linear relationship with a slope of 12.81 T/μ_B_. The hyperfine field increases with the magnetic moment increasing, as expected.

The average hyperfine field of one iron carbide is defined as the sum of the B_hf_ of various types of Fe multiplied by the weight of occupation ratio. The C/Fe ratio of a set of iron carbides increases from α-Fe (C/Fe = 0) to γ'-FeC (C/Fe = 0). Fig. 4(b) shows the dependency of the average B_hf_ of iron carbides on C/Fe ratio. It indicates that the average values of B_hf_ decrease linearly as the C/Fe ratio increases. For instance, α-Fe (C/Fe = 0) has the highest B_hf_ of 33.1 T, the B_hf_ of η-Fe_2_C with C/Fe = 0.5 is 17.99 T, and the B_hf_ of γ'-FeC with C/Fe = 1 decreases to 0 T. Furthermore, the hyperfine fields of Fe atoms also are influenced, especially in the neighborhood of C atoms. B_hf_ as a function of the number of C atoms in the neighborhood of a single Fe atom is shown in Fig. 4(c). It is found that the more C atoms in the neighborhood of a single Fe atom, the lower the hyperfine field. A similar trend for iron borides and nitrides is also reported by Le Caer *et al.*^29^.

The Bader charge analysis^42^,^43^,^44^ is implemented to Fe atoms and C atoms of various iron carbides. The average valence electrons of iron carbides are calculated and plotted as function of the C/Fe ratio in Fig. 4(d). It is found that the average valence electrons of Fe atoms decrease with increasing C/Fe ratio. To put it another way, the Fe atoms of iron carbide with a higher C/Fe ratio transfer more electrons to C atoms. For iron alloy systems and compounds, the Fermi contact field is the main contribution to hyperfine field, and has its origin in a different density of s-like electrons with spin-up and spin-down within the volume of the nucleus^41^. Therefore, the average hyperfine field will decrease if the different spin density decreases.

## Conclusions and Remarks

In this work, we employed DFT with GGA methods to investigate the relative stability and structural properties of pure iron phases and various iron carbides. Their hyperfine parameters were further predicted using the all-electron full-potential linearized augmented plane wave (FLAPW) with different exchange-correlation functionals. To further confirm the predictions, the mainly single-phase χ-Fe_5_C_2_ and θ-Fe_3_C were synthesized and the experimental Mössbauer spectra were acquired in the range from 13 K to 298 K.

Compared with experimental data, we pointed out that GGA-PBEsol accurately described the hyperfine parameters of α-Fe, χ-Fe_5_C_2_, and θ-Fe_3_C. Based on this reliable approach, Mössbauer parameters and spectrum of γ'-FeC, η-Fe_2_C, ζ-Fe_2_C, h-Fe_7_C_3_, o-Fe_7_C_3_, γ'-Fe_4_C, γ''-Fe_4_C, and α'-Fe_16_C_2_ were predicted by using GGA with PBEsol.

A relationship between magnetic properties and structure was proposed. A linear relationship between B_hf_ and μ_B_ with a slope of 12.81 T/μ_B_ was found, and the proportionality constant may vary from system to system. The relationship obtained provided the design strategy for Fe-based catalysts and materials.

We pointed out that the computed Mössbauer parameters from the reliable approach can be treated as references to identify phases in future experiments and provide the fitting/reference database for experiments (which can improve the characterization techniques and instruments). In the meantime, we provided the benchmark information to develop new theoretical methods for strongly correlated metal systems.

## Methods

### Syntheses

α-Fe_2_O_3_ used in present study was prepared by a combination of precipitation and spray-drying technologies. In brief, a solution containing Fe(NO_3_)_3 _• 9H_2_O was used in precipitation with NH_4_OH solution as a precipitator at pH = 8.5~9.0 and *T* = 70 °C. The precipitate was washed and then filtered. The mixture was reslurried and spray-dried. Finally, a sample with diameters of 20~26 μm was calcined at 450 °C for 5 h in a muffle furnace.

Samples of χ-Fe_5_C_2_ and θ-Fe_3_C were prepared using the gas carburization method. First, the precursor α-Fe_2_O_3_ was reduced to α-Fe by H_2_ at 300 °C for 24 h (99.999%, 80 ml/min). The sample of ε-carbide was carburized by 1:4 CO/H_2_ at 180 °C for 48 h and χ-Fe_5_C_2_ was synthesized by decomposition of ε-carbide annealing under Ar (99.999%, 50 ml/min) at 350 °C. θ-Fe_3_C was obtained by directly carburization of α-Fe under H_2_/CO = 30, at 460 °C for 48 h.

### Mössbauer Spectroscopy Measurement and Analysis

The Mössbauer spectrums of the synthesized χ-Fe_5_C_2_ and θ-Fe_3_C were acquired in an MR-351 constant-acceleration Mössbauer spectrometer (FAST, Germany) drive with a triangular reference signal at the temperature range of 13–298 K. The radioactive source was a ^57^Co source in a Rh matrix. Data analysis was performed using the MossWinn software package with the evolution algorithm^45^. The spectra were modeled as a combination of singlets, quadruple doublets, and magnetic sextets based on a Lorentzian line shape profile. Isomer shift values reported in this work are relative to that of α-Fe foil at room temperature.

### Iron Carbide Optimizations

The geometry optimization of iron carbides and α-Fe were performed using a plane-wave periodic DFT method as implemented in the Vienna *ab initio* simulation package (VASP)^46^,^47^. The wavefunction was expanded in a plane wave basis set and electron-ion interaction was described by the projector augmented wave (PAW) method^48^,^49^ with energy cutoff of 400 eV. For the treatment of electron exchange and correlation, the generalized gradient approximation of the Perdew-Burke-Ernzerhof scheme (GGA-PBE)^50^ was used. The semi-core p states were treated as valence with 14 valence electrons (3p3d^7^4s^1^) in the iron-potential, and the carbon-potential had 4 valence electrons (2s^2^2p^2^). Many previous studies^5^,^6^,^7^,^10^ have proved that GGA-PBE yields reasonable structural parameters and electronic properties of iron and iron carbides. A second-order Methfessel-Paxton^51^ electron smearing with σ = 0.2 eV was used to ensure accurate energies with errors due to smearing of less than 1 meV per unit cell. The Brillouin zone was sampled with the Monkhorst-Pack scheme. Due to the ferromagnetic nature of iron carbides and α-Fe, spin polarization was included in all calculations. The convergence criteria for structural optimizations and energy calculations were set to high quality with the tolerance for force and energy of 0.02 eV/Å and 10^−5 ^eV, respectively. The phonon calculations were performed within the harmonic approximation using the direct method based on the calculated non-vanishing Hellman-Feynman forces employing the Phonopy code^52^ to obtain the formation energy with zero point energy corrections of iron carbides.

Calculations for the α-Fe bulk structure with a k-point mesh of (13 × 13 × 13) give a lattice constant of 2.832 Å and a magnetic moment of 2.13 μ_B,_ which are in very good agreement with other DFT calculations and experiment^53^. In the calculations of iron carbides, we used (5 × 7 × 9), (5 × 5 × 5), (11 × 11 × 11), (7 × 5 × 3), (5 × 7 × 7), and (9 × 7 × 9) k-meshes for η-Fe_2_C, ζ-Fe_2_C, h-Fe_7_C_3_, o-Fe_7_C_3_, χ-Fe_5_C_2_, and θ-Fe_3_C, a (7 × 7 × 7) mesh of special k-points for γ'-FeC, γ'-Fe_4_C, γ''-Fe_4_C, and α'-Fe_16_C_2_, respectively. The calculated lattice parameters of iron carbides are listed in Table S1. One can see that the structural parameters of various iron carbides are very close to previous theoretical and experimental studies^5^,^8^ (error < 2.2%).

### Hyperfine Parameter Calculations

The calculations of the Mössbauer parameters of α-Fe and various iron carbides used the Wien2k package^54^,^55^ using the full-potential (linearized)-augmented-plane-wave plus local orbitals method^56^ to solve the Kohn-Sham equations. We used a separation energy of −7.0 Ry to distinguish between core and valence electron states. For all the iron carbides the muffin-tin radii (R_MT_) of 1.89–2.2 a.u. for Fe, and 1.54–1.6 a.u. for C were used respectively. The size of the basis set is given by *R*_*mt*_*K*_*max*_ = 7.0 and the maximum *l* quantum number *l*_*max*_ = *10*. The number of k points in the whole Brillouin zone was set to 10000. In addition, spin polarization and spin-orbit coupling were taken into account in the iron carbides. The convergence criteria for all calculations was set to high quality with the tolerance for charge of 0.0001 e. LDA, GGA-PBE, GGA-PBEsol, meta-GGA, GGA + U, and hybrid functionals were used to deal with the exchange-correlation potential.

The isomer shift originates from the coulomb interaction between the nuclear charge and the electronic density at the nucleus, which provides the information of the nucleus structure. It can be expressed using the formula IS = α(ρ_0_−ρ_reference_), where α is a nuclear constant, ρ_0_ is the electronic density at nucleus position of a sample, and ρ_reference_ is the electronic concentration present at the nucleus of α-Fe. As shown in Table S3, many researchers have calculated the charge density at the nucleus, and suggested the values of the calibration constant α for ^57^Fe. The value of the electron density at the nucleus in α-iron is seriously influenced by the basis set chosen and the relativistic effect. For instance, the value of the electron density at the nucleus of FeF_6_^3−^ without the relativistic effect is of 11615.363 a.u.^−3 ^57, which is smaller than the value of 15066.82 a.u.^−3^ with considering the relativistic effect by Freeman^58^. Our result (15310.102 a.u.^−3^) is in very good agreement with Freeman’s work and other works using the full-potential (linearized)-augmented-plane-wave plus the relativistic effect. It should be noted that the calculated isomer shift values are dependent upon the electron density difference at the nucleus between samples and α-iron and the calibration constant α. Duff calculated the electron density of the cluster model of FeF_6_^3−^ using an unrestricted Hartree-Fock method and determined α to be −0.23 a.u.^3^ mm/s^57^. However, Wdowik *et al.* indicated that previous studies did not consider solid-state effects on the calculation of α. The value of α was calibrated to be −0.291 a.u.^3^ mm/s by using full-potential linearized augmented plane-waves^59^. In this work, we prefer to use the value of −0.291 a.u.^3^ mm/s, since iron carbides are in solid state, and similar to solid Fe halides studied by Wdowik *et al.*

The quadrupole splitting (QS) reflects the interaction between the nuclear energy levels and surrounding electric field gradient (EFG). The electric field gradient (EFG) is expressed by its main component (V_zz_), and the QS value can be calculated using QS = eQV_zz_(1 + η^2^)^1/2^, where η is the asymmetry parameter, e and Q denote one proton charge and the nuclear electric quadrupole moment of the excited state of ^57^Fe with nuclear-spin quantum number I > 1, respectively. In this work we used the value Q = 0.16 × 10^−28^ m^2^, which was determined by Dufek *et al.*^60^.

## Additional Information

**How to cite this article**: Liu, X.-W. *et al.* Mössbauer Spectroscopy of Iron Carbides: From Prediction to Experimental Confirmation. *Sci. Rep.* **6**, 26184; doi: 10.1038/srep26184 (2016).

## Supplementary Material

Supporting Information

## Figures and Tables

**Table 1:** The calculated and experimental isomer shift (IS, mm/s), quadrupole splitting (QS, mm/s), hyperfine fields (B_hf_ in Tesla) and magnetic moment (Mag, μ_B_) of α-Fe, χ-Fe_5_C_2_, and θ-Fe_3_C within the various functionals.

	exp. (0K)	hybrid-HF			GGA + U (U in eV)			meta-GGA	GGA		LDA
		α = 0.25	α = 0.1	α = 0.01	2.5	3.5	4.5	TPSS	PBE	PBEsol	
Fe											
QS		−0.16	−0.22	0.02				0.00	0.00	0.00	0.00
B_hf_	−33	−45.2	−44.7	−32.7	−22.1	−16.4	−8.71	−31.2	−31.8	−33.5	−30.4
Mag	2.2	2.98	2.93	2.31	1.98	1.85	1.65	2.22	2.24	2.25	2.22
θ-Fe_3_C											
IS(Fe1)	0.33	0.93	0.39	0.18	0.21	0.22	0.22	0.19	0.21	0.28	0.25
QS(Fe1)	0.00	0.05	0.02	0.02	0.02	0.03	0.05	0.02	0.02	0.02	0.02
B_hf_(Fe1)	−25.3	−32.3	−30.9	−24.7	−23.4	−22.8	−9.3	−23.1	−23.8	−23.7	−20.7
Mag(Fe1)		2.64	2.26	1.94	1.89	1.87	1.06	1.87	1.91	1.86	1.68
IS(Fe2)	0.33	0.67	0.40	0.18	0.20	0.22	0.22	0.19	0.19	0.27	0.25
QS(Fe2)	0.03	−0.03	0.06	0.05	0.04	0.01	0.06	0.05	0.05	0.05	0.045
B_hf_(Fe2)	−26.6	−38.2	−31.8	−25.4	−23.4	−22.5	−4.1	−23.8	−24.4	−24.4	−21.8
Mag(Fe2)		2.74	2.41	2.02	1.97	1.93	0.26	1.95	1.98	1.95	1.83
χ-Fe_5_C_2_											
IS(Fe1)	0.39	0.51	0.46	0.26	0.29	0.31	0.32	0.27	0.28	0.36	0.33
QS(Fe1)	0.12	−0.06	0.06	0.05	0.05	0.03	0.07	0.05	0.05	0.05	−0.05
B_hf_(Fe1)	−26.1	−38.5	−32.3	−24.9	−22.8	−21.9	−11.4	−22.8	−23.5	−23.7	−21.3
Mag(Fe1)		2.74	2.48	2.13	2.12	2.04	0.71	2.09	2.11	2.08	1.99
IS(Fe2)	0.35	0.41	0.36	0.16	0.19	0.20	0.19	0.18	0.17	0.25	0.23
QS(Fe2)	0	0.05	0.03	0.03	0.04	0.04	0.05	0.03	0.03	0.03	−0.03
B_hf_(Fe2)	−22.7	−32.9	−28.9	−22.3	−21.0	−20.4	−0.7	−20.4	−21.0	−20.9	−18.4
Mag(Fe2)		2.54	2.22	1.73	1.72	1.73	0.50	1.65	1.69	1.64	1.51
IS(Fe3)	0.33	0.43	0.39	0.18	0.21	0.22	0.21	0.20	0.19	0.26	0.24
QS(Fe3)	0	−0.03	0.02	0.02	0.02	0.01	0.04	0.02	0.02	0.02	−0.02
B_hf_(Fe3)	−13.8	−24.1	−20.8	−15.2	−12.6	−9.8	−5.5	−13.7	−14.1	−14.0	−12.4
Mag(Fe3)		2.33	1.77	1.13	0.95	0.85	0.70	1.03	1.07	1.03	0.97

(The negative sign indicates that the hyperfine field is in the opposite direction to the magnetic moment).

**Table 2:** Relative error (%) of IS (mm/s) and B_hf_ (T) of Fe, θ-Fe_3_C, and χ-Fe_5_C_2_.

Parameters	HF	meta-GGA	GGA		LDA
	α = 0.01	TPSS	PBE	PBEsol	
Fe					
Bhf	−0.9	−5.5	−3.6	1.5	−7.9
θ-Fe_3_C					
IS(Fe1)	−45.5	−42.4	−36.4	−15.2	−24.2
B_hf_(Fe1)	−2.4	−8.7	−5.9	−6.3	−18.2
IS(Fe2)	−45.5	−42.4	−42.4	−18.2	−24.2
B_hf_(Fe2)	−4.5	−10.5	−8.3	−8.3	−18.0
χ-Fe_5_C_2_					
IS(Fe1)	−33.3	−30.8	−28.2	−7.7	−15.4
B_hf_(Fe1)	−4.6	−12.6	−10.0	−9.2	−18.4
IS(Fe2)	−54.3	−48.6	−51.4	−28.6	−34.3
B_hf_(Fe2)	−1.8	−10.1	−7.5	−7.9	−18.9
IS(Fe3)	−45.5	−39.4	−42.4	−21.2	−27.3
B_hf_(Fe3)	10.1	−0.7	2.2	1.4	−10.1

**Table 3:** The theoretical Mössbauer parameters (IS (mm/s), QS (mm/s), and B_hf_ (T)) and magnetic moment (Mag, μ_B_) of γ′-FeC, η-Fe_2_C, ζ-Fe_2_C, h-Fe_7_C_3_, o-Fe_7_C_3_, γ′-Fe_4_C, γ″-Fe_4_C, and α′-Fe_16_C_2_.

	γ′-FeC	η-Fe_2_C	ζ-Fe_2_C	h-Fe_7_C_3_	o-Fe_7_C_3_	γ′-Fe_4_C	γ′′-Fe_4_C	α′-Fe_16_C_2_
IS(Fe1)	0.42	0.29	0.30	0.30	0.34	0.28	0.12	0.00
QS(Fe1)	0.00	−0.04	−0.04	−0.06	−0.05	0.00	−0.10	−0.05
B_hf_(Fe1)	0.00	−18.0	−18.3	−24.8	−22.9	−34.5	−28.4	−26.9
Mag(Fe1)	0.00	1.67	1.68	1.98	1.90	2.96	2.01	2.03
IS(Fe2)				0.22	0.29	0.24		0.15
QS(Fe2)				0.04	0.05	−0.09		−0.04
B_hf_(Fe2)				−15.9	−18.7	−26.2		−28.4
Mag(Fe2)				1.39	1.68	1.71		2.18
IS(Fe3)				0.20	0.28	0.24		0.18
QS(Fe3)				0.04	−0.03	−0.09		−0.01
B_hf_(Fe3)				−20.0	−22.9	−26.2		−35.1
Mag(Fe3)				1.62	1.70	1.71		2.69
QS(Fe4)					0.22			
B_hf_(Fe4)					0.05			
Mag(Fe4)					−19.9			
IS(Fe5)					1.66			
QS(Fe5)					0.22			
B_hf_(Fe5)					0.04			
Mag(Fe5)					−17.4			
QS(Fe5)					1.46			

The negative sign of B_hf_ indicates that the hyperfine field is in the opposite direction to the magnetic moment.
