# Supplementary material for: Mössbauer Spectroscopy of Iron Carbides: From Prediction to Experimental Confirmation
Source: Sci Rep. 2016 May 18;6:26184. doi: 10.1038/srep26184 (PMC4870625; doi:10.1038/srep26184)
Supplement: Supporting Information [file srep26184-s1.pdf]

Supporting Information

Mössbauer Spectroscopy of Iron Carbides: From Prediction to Experimental Confirmation

Xing-Wu Liu,[1,2,3] Shu Zhao,[1,2,3] Yu Meng,[1,2,3] Qing Peng,[4] Albert Dearden,[5] Chun-Fang Huo,[1,2*] Yong Yang,[1,2] Yong-Wang Li,[1,2*] & Xiao-Dong Wen,[1,2*]

[1]*State Key Laboratory of Coal Conversion, Institute of Coal Chemistry, Chinese Academy of Sciences, Taiyuan, 030001, P.R. China;*

[2]*National Energy Center for Coal to Clean Fuels, Synfuels China Co., Ltd, Huairou District, Beijing, 101400, P.R. China.*

[3]*University of Chinese Academy of Sciences, No.19A Yuquan Road, Beijing, 100049, P.R .China.*

[4]*Department of Mechanical, Aerospace and Nuclear Engineering, Rensselaer Polytechnic Institute, Troy, NY 12180, USA*

[5]*Department of Physics, Berea College, Berea, KY 40403, USA*

*: Correspondence and requests for materials should be addressed to X.D.W. (email: wxd@sxicc.ac.cn) or Y.W.L. (email: ywl@sxicc.ac.cn) or C.F.H. (email: huochunfang@synfuelschina.com.cn)

Contents

Table S1. A summary of the structural parameters of iron carbides**.** 3

Table S2 Comparison Mössbauer parameters of θ-Fe_3C and χ-Fe_5C_2 at various temperature with previous work (Le Caer, G. *et al. J. Solid State Chem.* **1976**, *19*, 19.) 5

Table S3. Method used, isomer shift calibration constant α, and charge density at the nucleus. 7

Table S4. Magnetic moment (μ_B), Valence electron (VE) and Coordination atoms for individual Fe atoms of iron carbides within the distance lower than 2.7 Å. 8

Figure S1. Heat of reaction for (x/y)Fe + 2CO = (1/y)Fe_xC_y + CO_2. 4

Figure S2. Histograms of various iron carbides. (a) γ'-FeC, (b) η-Fe_2C,(c) ζ-Fe_2C, (d) χ-Fe_5C_2, (e) h-Fe_7C_3, (f) o-Fe_7C_3, (g) θ-Fe_3C, (h) ε-Fe_3C, (i) γ'-Fe_4C, (j) γ''-Fe_4C, and (k) α'-$Fe_{16}C_2$ 9

Figure S3. Histogram of Fe-C bond lengths in ICSD 10

Table S1. A summary of the structural parameters of iron carbides.

Formula	Space group	Present work	Lattice constants	Atom site occupancy
γ'-FeC	FM-3M (225)	a = b = c = 3.99<br>α = β = γ = 90°	a = b = c = 4.08, [1,2]<br>α = β = γ = 90°	Fe1 4a (0.0 0.0 0.0)<br>C1 4b (0.5 0.5 0.5)
η-Fe_2C	Pnnm (58)	a = 4.71, b = 4.26<br>c = 2.81<br>α = β = γ = 90°	a = 4.70, b = 4.32,<br>c = 2.83[3]<br>α = β = γ = 90°	Fe1, 4g (0.6667, 0.25, 0)<br>C1, 2a (0, 0, 0)
ζ-Fe_2C	Pbcn (60)	a = 4.29, b = 5.46,<br>c = 4.84<br>α = β = γ = 90°	a = 4.30, b = 5.48,<br>c = 4.85[4]<br>α = β = γ = 90°	Fe1, 8d (0.249, 0.616, 0.579)<br>C1, 4c (0.000, 0.364, 0.750)
h-Fe_7C_3	$P6_3mc$ (186)	a = b = 6.806,<br>c = 4.493<br>α = β = 90°,γ = 120°	a = b = 6.882,<br>c = 4.540, [5]<br>α = β = 90°,γ = 120°	Fe1, 2b (0.333, 0.666, 0.818)<br>Fe2, 6c (0.456, 0.543, 0.318)<br>Fe3, 6c (0.122, 0.878, 0.000)<br>C1, 6c (0.187, 0.813, 0.580)
o-Fe_7C_3	Pnma (62)	a = 4.510, b = 6.840,<br>c = 11.700<br>α = β = γ = 90°	a = 4.537, b = 6.892,<br>c = 11.913<br>α = β = γ = 90°	Fe1, 8d (0.25, 0.07, 0.02)<br>Fe2, 8d (0.00, 0.07, 0.81)<br>Fe3, 4c (0.24, 0.25, 0.20)<br>Fe4, 4c (0.25, 0.25, 0.42)<br>Fe5, 4c (0.00, 0.25, 0.63)<br>C1, 8d (0.11, 0.03, 0.35)<br>C2, 4c (0.38, 0.25, 0.57)
χ-Fe_5C_2	C2/c (15)	a = 11.559, b = 4.494,<br>c = 4.979,<br>α = γ = 90°, β = 97.60°	a = 11.588, b = 4.579,<br>c = 5.059, [6]<br>α = γ = 90°, β = 97.75°	Fe1, 8f (0.097, 0.078, 0.423)<br>Fe2, 8f (0.215, 0.581, 0.306)<br>Fe3, 4e (0, 0.561, 0.25)<br>C1, 8f (0.107, 0.285, 0.149)
θ-Fe_3C	Pnma (62)	a = 5.025, b = 6.726,<br>c = 4.471<br>α = β = γ = 90°	a = 5.089, b = 6.744,<br>c = 4.525, [7]<br>α = β = γ = 90°	Fe1, 8d (-0.186, 0.563, -0.328)<br>Fe2, 4c (0.036, 0.250, 0.852)<br>C1, 4c (0.890, 0.250, 0.450)
γ''-Fe_4C	PM-3m (221)	a = b = c = 3.757<br>α = β = γ = 90°		Fe1, 1a (0.0 0.0 0.0)<br>Fe2, 3c (0.5 0.5 0.0)<br>C1, 1b (0.5 0.5 0.5)
γ'-Fe_4C	P-43m (215)	a = b = c = 3.829<br>α = β = γ = 90°	a = b = c = 3.878, [8]<br>α = β = γ = 90°	Fe1, 4e (0.265, 0.265, 0.265)<br>C1, 1a (0.0, 0.0, 0.0)
α'-$Fe_{16}C_2$	I4/mmm (139)	a = b =5.641, c = 6.259<br>α = β = γ = 90°	a = b = 5.655, c = 6.262<br>α = β = γ = 90° [9]	C1, 2a (0.000, 0.000, 0.000)<br>Fe1, 4e (0.000, 0.000, 0.707)<br>Fe2, 8h (0.756, 0.244, 0.000)<br>Fe3, 4d (0.000, 0.500, 0.750)

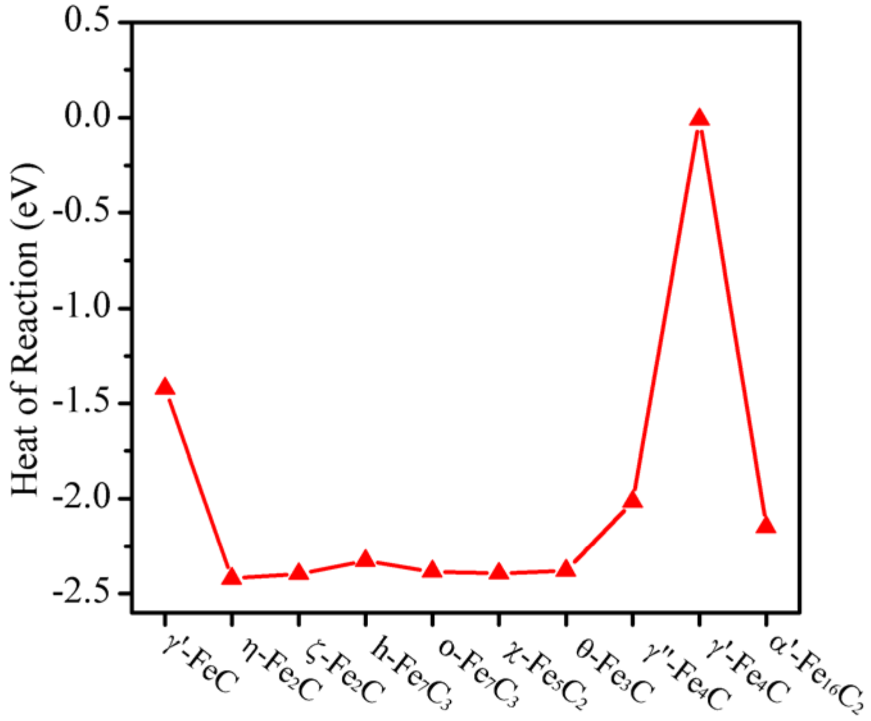

Figure S1. Heat of reaction for (x/y)Fe + 2CO = (1/y)Fe_xC_y + CO_2.

Table S2 Comparison Mössbauer parameters of θ-Fe_3C and χ-Fe_5C_2 at various temperature with previous work (Le Caer, G. *et al. J. Solid State Chem.* **1976**, *19*, 19.)

		Present work						Previous work		
		13K	77K	130K	190K	245K	298K	4K	77K	298K
Fe_3C										
Fe1	IS(mm/s)	0.33	0.33	0.20	0.28	0.18	0.16	0.32	0.31	0.18
		(4.5)	(7.1)				(-11)			
	B_{hf}(T)	25.1	24.8	24.4	23.6	22.7	20.2	24.9	24.6	20.8
		(0.6)	(0.7)				(-3.0)			
	QS(mm/s)	0.00	0.01	0.01	0.01	0.01	0.01	0.01	0.01	0.02
	Area(%)	66.7	65.8	66.4		62.8	63.4			
Fe2	IS(mm/s)	0.33	0.34	0.20	0.29	0.16	0.09	0.32	0.31	0.18
		(3.7)	(11)							
	B_{hf}(T)	26.4	26.1	25.6	24.8	24.0	21.0	24.7	24.5	20.6
		(6.8)	(6.6)				(2.1)			
	QS(mm/s)	0.03	0.03	0.03	0.02	0.04	0.06	0.01	0.01	0.02
	Area(%)	33.3	34.2	33.6		31.4	31.7			
Spm Fe	IS(mm/s)					0.17	0.20			
	QS(mm/s)					1.12	0.96			
	Area(%)					5.8	4.8			
χ-Fe_5C_2										
Fe1	IS(mm/s)	0.39	0.39	0.38	0.35	0.33	0.14	0.38	0.37	0.23
		(2.8)	(4.7)				(-38.6)			
	B_{hf}(T)	25.8	25.7	25.3	24.7	24.11	21.5	25.2	24.8	21.6
		(2.5)	(3.6)				(-0.3)			
	QS(mm/s)	0.12	0.13	0.12	0.11	0.11	0.09	0.08	0.08	0.04
	Area(%)	31.0	31.9	35.9	33.1	34.8	34.6			
Fe2	IS(mm/s)	0.35	0.33	0.32	0.30	0.28	0.15	0.33	0.30	0.2
		(6.9)	(9.5)				(-23.7)			
	B_{hf}(T)	22.5	22.3	21.9	21.2	20.7	18.3	22	21.7	18.5
		(2.4)	(2.6)				(-1.1)			
	QS(mm/s)	-0.01	0.00	0.00	-0.01	0.02	0.07	0.12	0.10	0.05
	Area(%)	31.0	32.0	38.4	33.1	37.8	36.1			
Fe3	IS(mm/s)	0.33	0.33	0.32	0.30	0.28	0.10			
	B_{hf}(T)	13.5	13.6	13.2	12.8	12.3	10.8	13.4	12.6	11.2
		(0.9)	(7.9)				(-3.3)			
	QS(mm/s)	-0.09	0.00	0.00	-0.01	0.01	0.12			
		15.5	16.0	16.9	16.6	17.4	21.3			
Fe_3O_4(A)	IS(mm/s)	0.50	0.48	0.40						
	B_{hf}(T)	49.5	47.0	50.0	49.9					
	QS(mm/s)	0.00	-0.01	-0.07	-0.12					
	Area(%)	22.4	20.1	8.8	7.2					
Spm Fe	IS(mm/s)			0.35	0.38	0.31	0.15			

QS(mm/s)	1.45	1.32	1.14	1.02
Area(%)	2.32	10.0	10	10.3

Table S3. Method used, isomer shift calibration constant α, and charge density at the nucleus.

Method			α (a.u.^{3} mm/s)	Charge density at the nucleus (a.u.$^{-3}$)	Reference
Gaussian type basis set	nonrelativistic	unrestricted HF	-0.23	(FeF_6^{3-})[h] 11615.363	Duff[a]
		HF	-0.24	11619.949	Nieuwpoort[b]
		HF (6-311G)	-0.266	(FeF_3)[h] 11619.84	Oldfield[c]
		BPW91 (6-311G)	-0.381	(FeF_3)[h] 11620.14	Oldfield[c]
		B3LYP (6-311G)	-0.323	(FeF_3)[h] 11615.74	Oldfield[c]
	relativistic	Huckel-type MO		(FeF_6^{3-})[h] 15066.82	Freeman[d]
		HF		14894.186	Filatov[e]
		MP2		14895.146	Filatov[e]
		CCSD(T)		14894.788	Filatov[e]
Full-potential linearized augmented plane-wave		LDA + U	-0.291	15322.046	Wdowik[f]
		PBE		15310.102	Abreu[g]
		PBE		15309.481	present work
		PBEsol		15275.312	present work
		GGA + U		15309.737	present work

a. Duff, K. J. *Phys. Rev. B* **1974**, *9*, 66.
b. Nieuwpoort, *et al. Phys. Rev. B* **1978**, *17*, 91.
c. Zhang, Y. *et al. J. Am. Chem. Soc* **2002**, *124*, 7829.
d. Trautwein, A. *et al.*P. *Phys. Rev. B* **1975**, *11*, 4101.
e. Filatov, M. *et al. J. Chem. Phys* **2007**, *127*, 084101.
f. Wdowik, U. D. *et al. Phys. Rev. B* **2007**, *76*, 155118.
g. Abreu, Y. *et al. Solid State Commun.* **2014**, *185*, 25.
h. In parentheses, the substance for calculation of the charge density inside the Fe nucleus

Table S4. Magnetic moment (μ_B), Valence electron (VE) and Coordination atoms for individual Fe atoms of iron carbides within the distance lower than 2.7 Å.

Species		Magnetic moment (μ_B)	VE (e)		Coordination
			Fe	C	
γ'-FeC	Fe1	0.00	13.15	4.85	6C
η-Fe_2C	Fe1	1.69	13.41	5.19	3C,2Fe
ζ-Fe_2C	Fe1	1.71	13.40	5.21	3C,1Fe
h-Fe_7C_3	Fe1	1.52	13.5	5.14	3C,6Fe
	Fe2	2.02	13.53		4C,9Fe
	Fe3	1.36	13.49		3C,7Fe
o-Fe_7C_3	Fe1	1.96	13.48	5.14	4C,7Fe
	Fe2	1.72	13.47	5.21	3C,5Fe
	Fe3	1.77	13.60		2C,8Fe
	Fe4	1.71	13.51		3C,6Fe
	Fe5	1.48	13.50		3C,4Fe
χ-Fe_5C_2	Fe1	2.12	13.54	5.14	2C,3Fe
	Fe2	1.66	13.58		3C,6Fe
	Fe3	0.99	13.48		4C, 3Fe
θ-Fe_3C	Fe1	1.84	13.60	5.19	3C,5Fe
	Fe2	1.97	13.61		2C,2Fe
γ''-Fe_4C	Fe1	3.05	13.71	5.35	2Fe
	Fe2	1.75	13.65		2C,4Fe
γ'-Fe_4C	Fe1	2.11	13.78	4.89	1C,3Fe
α'-$Fe_{16}C_2$	Fe1	2.1	13.9	5.19	1C,9Fe
	Fe2	2.24	13.83		1C,8Fe
	Fe3	2.79	13.85		8Fe
α-Fe	Fe1	2.2	14.00	4.85	8Fe

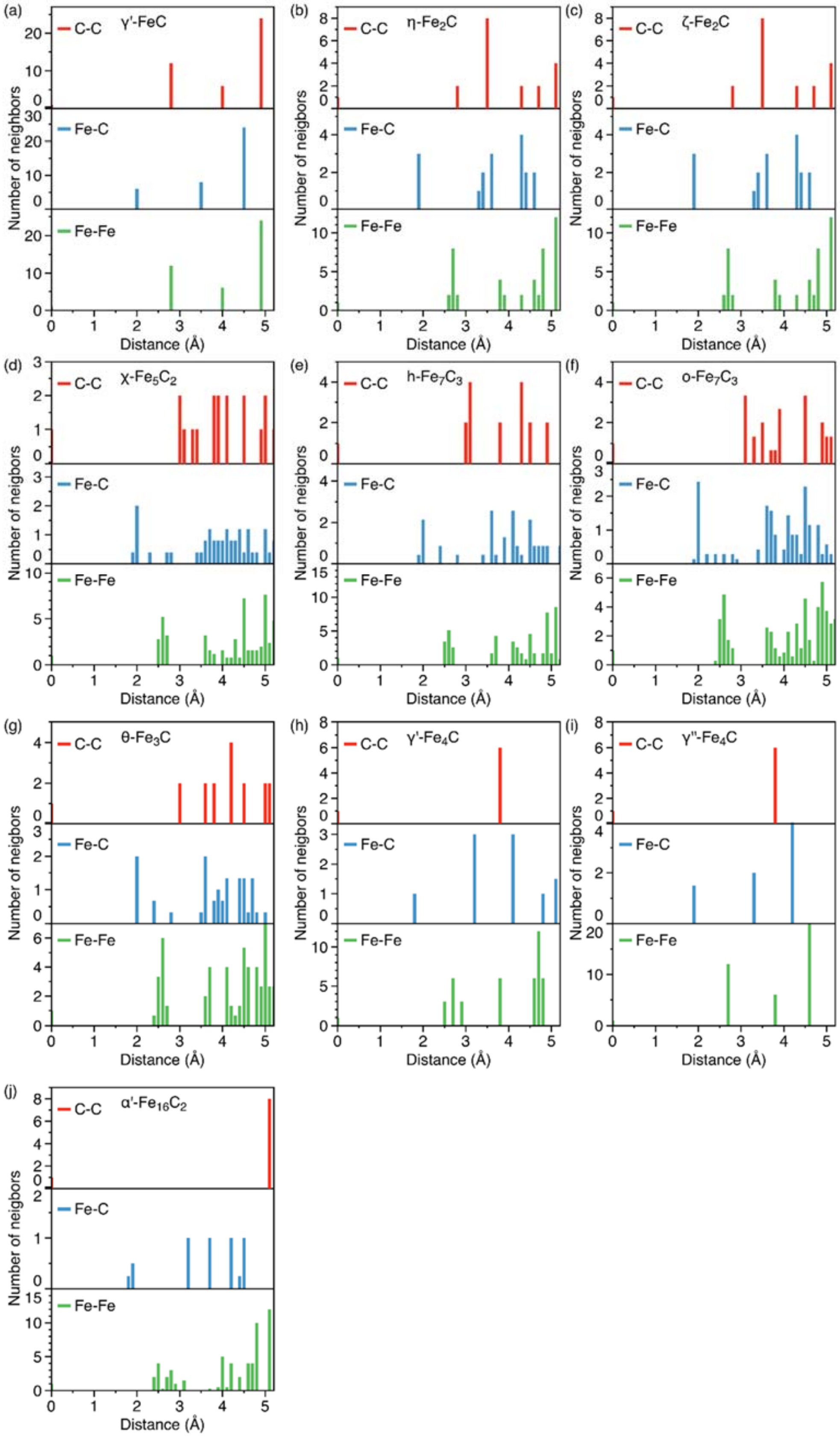

Figure S2. Histograms of various iron carbides. (a) γ'-FeC, (b) η-Fe_2C,(c) ζ-Fe_2C, (d) χ-Fe_5C_2, (e) h-Fe_7C_3, (f) o-Fe_7C_3, (g) θ-Fe_3C, (h) ε-Fe_3C, (i) γ'-Fe_4C, (j) γ''-Fe_4C and (k) α'-$Fe_{16}C_2$

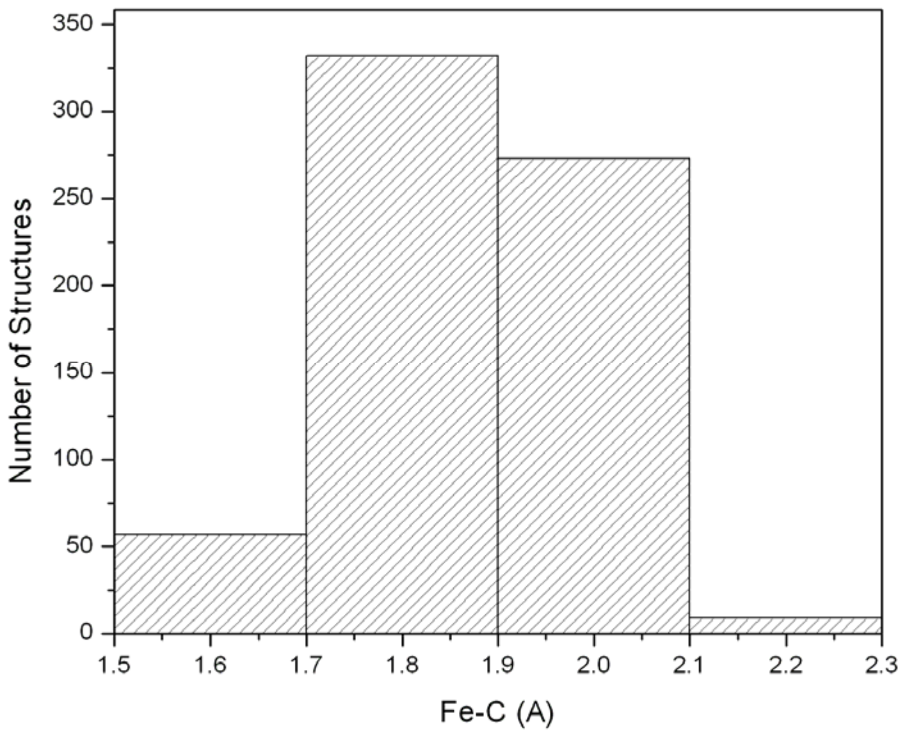

Figure S3. Histogram of Fe-C bond lengths in ICSD

References

(1) Lee, B.-J. Acta Materialia, **2006**, 56 701–711.

(2) Cusenza, S.; Schaaf, P. Mater. Lett. **2009**, 63, 1445–1447.

(3) Hirotsu, Y.; Nagakura, S. Acta Metallurgica, **1972**, 20, 645-655.

(4) Fang, C. M.; van Huis, M. A.; Jansen, J.; Zandhergen, H. W. Phys. Rev. B **2011**, 84, 094102

(5) Herbstein, E. H.; Snyman, J. A. Inorganic Chem. **1964**, 3, 894-896.

(6) Retief, J. J. Power Diffraction, **1999**, 14, 130-132.

(7) Fasiska, E. J. Jeffrey, G. A. Acta Crystallographica, **1965**, 19, 463-471.

(8) Pinsker, Z. G.; Kaverin, S. V. Kristallografiya, **1956**, 1, 66-72.

(9) Fang, C. M.; van Huis, M. A.; Jansen, J.; Zandhergen, H. W. Phys. Rev. B **2012**, 85, 054116
